# The role of water in Earth's mantle

**DOI:** 10.1093/nsr/nwz071

**Published:** 2019-06-11

**Authors:** Eiji Ohtani

**Affiliations:** Graduate School of Science, Tohoku University, Sendai 980-8578, Japan

**Keywords:** water, hydrous phase, subducting slab, transition zone, lower mantle, hydrogen-bond symmetrization, core–mantle boundary

## Abstract

Geophysical observations suggest that the transition zone is wet locally. Continental and oceanic sediment components together with the basaltic and peridotitic components might be transported and accumulated in the transition zone. Low-velocity anomalies at the upper mantle–transition zone boundary might be caused by the existence of dense hydrous magmas. Water can be carried farther into the lower mantle by the slabs. The anomalous Q and shear wave regions locating at the uppermost part of the lower mantle could be caused by the existence of fluid or wet magmas in this region because of the water-solubility contrast between the minerals in the transition zone and those in the lower mantle. δ-H solid solution AlO_2_H–MgSiO_4_H_2_ carries water into the lower mantle. Hydrogen-bond symmetrization exists in high-pressure hydrous phases and thus they are stable at the high pressures of the lower mantle. Thus, the δ-H solid solution in subducting slabs carries water farther into the bottom of the lower mantle. Pyrite FeO_2_H*_x_* is formed due to a reaction between the core and hydrated slabs. This phase could be a candidate for the anomalous regions at the core–mantle boundary.

## INTRODUCTION

Hydrogen is the most abundant element in the solar abundance. There are various modes of occurrence of hydrogen on Earth. Hydrogen exists as water vapor in the atmosphere, and water and ice in the ocean and land water, super-critical fluids in the volcanoes and Earth crusts, hydroxyls in hydrous and nominally anhydrous minerals in the crust and mantle, proton and hydroxyl (OH) in magmas, and hydrogen in metallic iron in the core.

Hydrogen and water play important roles in the dynamics of Earth's interior. They lower the internal friction of rocks and cause earthquakes, and dehydration embrittlement, namely the dehydration of hydrous minerals (such as serpentine) that causes fracturing. Water generates magmas by lowering the melting temperature of silicates in the mantle. Water softens rocks, namely water weakening, and enhances mantle convection.

The flux of water on Earth has been estimated by several authors. According to Peacock [1], the amount of water degassed to the surface through magmatism is 2 × 10^11^ kg/year. The water flux returned to the mantle by subducting slabs is ∼8.7 × 10^11^ kg/year. Thus, 6.7 × 10^11^ kg/year of water move to the deep interior associated with slab subduction. According to Wallace [2], there might be a balance in the flux of water between the input through subducting slabs and the output through degassing through arc volcanism to the surface, both with 3 × 10^11^ kg/year. On the other hand, van Keken *et al.* [3] estimated that a third of water, i.e. 7–10 × 10^11^ kg/year, penetrating through subduction is recycled into the mantle, whereas two-thirds of this water is degassed through dehydration of the slabs during subduction. In spite of uncertainties, it is important to specify the water reservoirs in the mantle, since a small amount of water can modify the properties of mantle materials.

The water contents were estimated by many procedures such as mineral inclusions in diamond, fluid or glass inclusions in magmas, mineral physics and the phase stability of the water-bearing system, and geophysical observations such as electrical conductivity and seismic observations. Reliable estimation of global water contents in the mantle is obtained by geophysical observations, since other procedures may represent the water contents in local regions, and the average water content may be different due to heterogeneity in the mantle. The water contents in Earth's interior estimated by geophysical observations are summarized in Table 1. Extensive studies have agreed that the transition zone is a major water reservoir, but the water storage in the lower mantle is poorly constrained. The water contents summarized in Table 1 indicate that the upper mantle, mantle transition zone and lower mantle contain 0.04, 0.2–1 and <2 oceans of water, respectively. Here, we discuss the various processes related to water in the upper mantle, transition zone, lower mantle and the core–mantle boundary, such as the water distribution, hydrous minerals and their O–H bonding nature, and the effects of water in the seismic anomalies and mantle dynamics.

**Table 1.** Summary of the water contents in the mantle deduced by geophysical observations.

Mantle zone	Water budget	Water content
Upper mantle	∼0.04 Ocean Mass	<100 ppm of water in normal mantle: Electrical conductivity [4]
		0.01 wt.% Electrical conductivity [5]
Mantle transition	0.2∼1 Ocean mass	0.1∼0.3 wt.% water in Pacific region based on electrical conductivity [5]
zone	(assuming Wet TZ 30%;	0∼ 0.1 wt.% water based on electrical conductivity [60]
	Dry TZ 70%)	0.1 wt.% water based on electrical conductivity [5]
		0.2∼2 wt.% water beneath Japan based on electrical conductivity and seismic tomography [61]
		0.5∼1 wt.% water beneath Pacific based on electrical conductivity and seismic tomography [22]
		< 0.1 wt.% water beneath Europe by electrical conductivity and seismic tomography [22]
		1 wt.% beneath western Pacific based on topography of the 410 and 660 km discontinuities [62]
Lower mantle	< 2 Ocean mass	> 0.1 wt.% beneath eastern Asia, oversaturated in water locally [30]
		<< 0.1 wt.% water in normal lower mantle [30]

## ROLE OF WATER IN THE UPPER MANTLE AND THE MANTLE TRANSITION ZONE

### Water in the upper mantle

The water content stored in the upper mantle estimated by various methods is shown in Table 1. Electrical conductivity data suggest the normal upper mantle is essentially dry and its water content is less than 100 ppm [4] or ∼100 ppm [5]. The average water content in olivine, orthopyroxene and clinopyroxene in the Udachinaya peridotite xenolith has been reported as 1–64 ppm [6], although a high water content 300 ppm of olivine due to metasomatism is reported in the same xenolith [7]. The water contents in the MORB (mid-oceanic ridge basalt) and OIB (ocean island basalt) source mantles are estimated to be 0.01 and 0.075 wt.%, respectively, based on the H_2_O/Ce ratio in basalt glasses [8]. These estimations indicate that the upper mantle is not homogeneous and the normal mantle represented by the depleted mantle of the MORB source is generally dry, with ∼100 ppm or less of water, whereas the OIB source mantle is wet, with water contents of ∼0.075 wt.%.

On the other hand, the water content in subducting slabs descending in the upper mantle is estimated to be 1–2 wt.% [3] based on the water content in hydrous phases. Thus, slabs can carry H_2_O into the mantle, which can affect geodynamics, such as seismicity, subduction processes, ascending plumes and magmatisms such as island arc volcanism, intraplate magmatism, hot spots and oceanic ridge magmatism. The average normal upper mantle is generally dry, as shown above, but the subducting slabs can carry a significant amount of water, in which two-thirds of the water dehydrates and generates magmas and triggers seismicity, whereas the rest can be stored in slabs and further carried into the deep mantle [3].

### A low-velocity region at the bottom of the upper mantle

**Figure 1.**
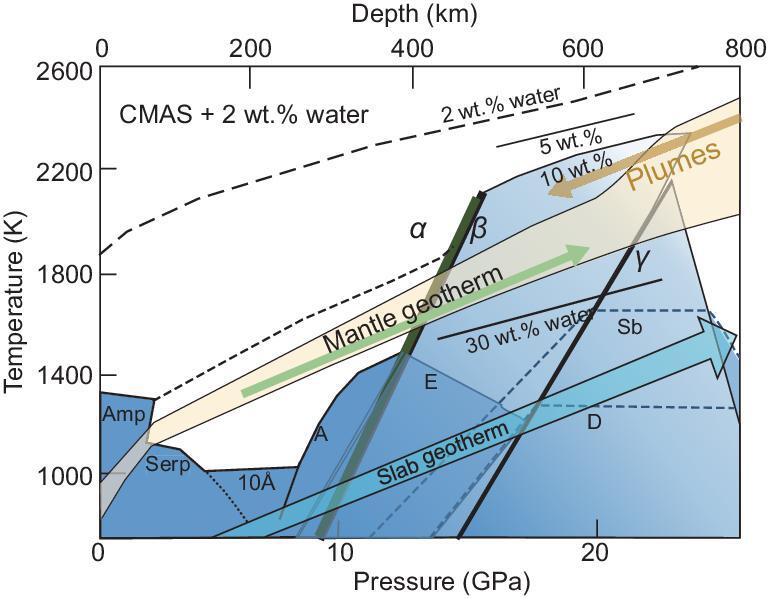
The melting and phase relations of hydrous mantle peridotite. The hydrous CMAS (CaO–MgO–Al_2_O_3_–SiO_2_) pyrolite [13] and hydrous CFMAS (CaO–FeO–MgO–Al_2_O_3_–SiO_2_) harzburgite [14] are shown in this figure. The solid lines are conditions of the melts containing 5–10 wt.% water formed by partial melting of the hydrous pyrolite at 2400–2500 K at 20 GPa [13] and the melt containing 30 wt.% water by partial melting of the hydrous harzburgite at 1800 K at 15∼20 GPa [14]. The dashed and dotted curves are the liquidus and solidus of pyrolite with 2 wt.% water, respectively. The yellow hatched area covers the uncertainty of the normal mantle geotherm and the green arrow is an average mantle-temperature profile and a blue arrow at lower temperatures shows the temperature profile of the slabs. There might be a thermal boundary at the depths of the mantle transition zone [35]. Thus, the temperature profile of the normal mantle shown as the yellow hatched area has a large uncertainty because of the complicated geodynamics due to convection patterns and chemical stratifications in this region [34]. Amp, amphibole; Serp, serpentine; 10 Å, 10 Å phase; A, phase A; E, phase E; Sb, superhydrous phase B;  α, olivine; β, wadsleyite; γ, ringwoodite. The thin dotted lines indicate the decomposition boundaries for superhydrous phase B (Sb) and phase D, and thin lines indicate the phase boundaries of serpenitine (Serp), 10 Å phase, phase A and phase E.

**Figure 2.**
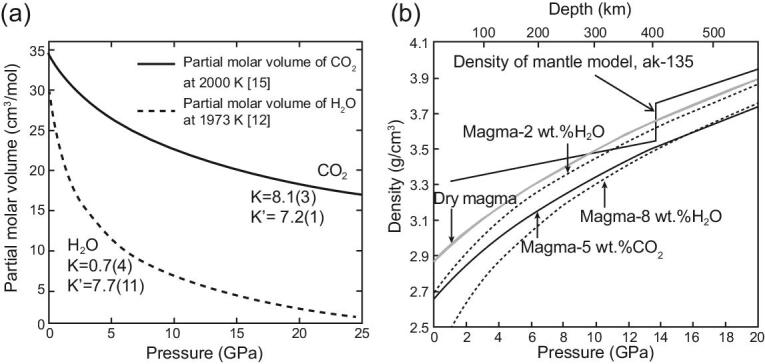
(a) Partial molar volumes of H_2_O and CO_2_ in magmas at high pressure and ∼2000 K [12,15]. The parameters for compression of the components are: V_0_ (partial molar volume at 0 GPa and 2000 K) = 36 cm^3^/mol, K_T0_ (isothermal bulk modulus) = 8.1(3) GPa, and K’ (its pressure derivative) = 7.2(1) for CO_2_, and V_0_ = 29.6 cm^3^/mol at 0 GPa and 1973 K, K_T0_ = 0.7(4), and K’ = 7.7(11) for H_2_O in magmas. (b) The compression curves of H_2_O and CO_2_-bearing peridotite magmas at high pressure and ∼1873 K, and density of the mantle model (ak-135; [67]). The gray curve is the density of a dry peridotite magma and the solid curve is the density of carbonated magma with 5 wt.% CO_2_ [15]. The dashed curves show the densities of magmas containing 2% and 8% water [12]. The mantle model ak-135 density is shown as a thin solid curve.

Seismological studies reported the existence of low-velocity regions at the bottom of the upper mantle (∼410-km depth) beneath Japan and north-east China [9], Europe [10] and the USA [11]. These regions might be caused by the existence of dense, volatile-rich magmas (e.g. [12]). Fig. 1 shows the phase and melting relations of wet peridotite at high pressure and temperature [13,14]. This figure shows a kink in the solidus curve near the 410-km discontinuity. This kink could be caused by the olivine–wadsleyite transformation with the water-solubility contrast between the two phases. Dehydration melting of plumes ascending from the lower mantle could occur at ∼410-km discontinuity. Fig. 2a and b shows the partial molar volumes of H_2_O and CO_2_ in magmas at high pressure and the density of the volatile-rich magmas [12,15]. The density of volatile-rich magmas has been studied by various authors using the *in situ* X-ray absorption method, sink-float method and *ab initio* calculations (e.g. [12,16]). Fig. 2a clearly indicates that H_2_O is more compressible compared to CO_2_ at high pressure and ∼2000 K, and a rapid densification of hydrous melts occurs at the pressure and temperature conditions representative of the base of the upper mantle, suggesting the possible existence of dense hydrous magmas. Fig. 2b shows the density of volatile-rich magmas at high pressure and 1873 K, which clearly indicates that the wet ultramafic magmas with the H_2_O content <5 wt.% is denser than the mineral assemblage at the bottom of the upper mantle, indicating the existence of gravitationally stable dense hydrous magmas along the plume geotherm.

### Hydrated mantle transition zone and existence of crust components

Recycling of the crustal components has been intensively studied in tectonics, geochemistry, petrology and geodynamics (e.g. [17,18]). The slabs are mainly composed of lithospheric components such as harzburgites, basalts, and continental and oceanic sediments [19].

Phase Egg, phase δ-AlO_2_H [20] and hydrous ringwoodite, namely spinel type of Mg_2_SiO_4_ containing water up to 1 wt.% [21], have been discovered as inclusions in diamonds. These observations suggest that the mantle transition zone is wet locally. Geophysical observations such as electrical conductivity and seismic tomography also suggest that the mantle transition zone is wet at least locally [22].

Continental and oceanic sediment components might be transported into the mantle transition zone. The fingerprints of sediment components have been reported from C and N isotopes of eclogitic diamond grains [23]. Hydrous minerals, such as phase Egg and phase δ-AlO_2_H stable under the transition-zone conditions [24,25] and TAPP (tetragonal almandine-pyrope phase) [26] might also be fingerprints of the crustal component included in diamond.

The crustal component subducted into the transition zone was modeled as a megalith by Ringwood [19]. The sediment component contains water as hydrous minerals such as phengite [27]. The existence of the component was suggested also by Kawai *et al.* [28]. The continental crustal materials can descend into the mantle transition zone, since silica minerals in this component transform to stishovite and the crustal materials become denser than the surrounding mantle materials [19]. Such continental crustal materials might play important roles as heat sources together with water carriers [27]: that is, the continental crustal materials contain a large amount of radiogenic elements such as K, U and Th, several hundred times higher than those of the surrounding mantle, and thus they may play a key role for heating the transition zone, upwelling and the generation of plumes [29].

## ROLE OF WATER IN THE LOWER MANTLE

### Origin of the low V_s_ and Q region at the top of the lower mantle

Lawrence and Wysession [30] reported the global attenuation pattern around the subducting slabs, indicating the lowest Q anomaly in the shallow lower mantle beneath Eastern Asia. Schmandt *et al.* [31] reported the existence of a low Q and low V_s_ region at the shallow lower mantle where volatile-rich magmas might be located. Recently, Sakamaki [32] argued a possibility that the dense hydrous magmas may exist at the bottom of the transition zone. A large compressibility of H_2_O in magmas reduces the volume of wet magmas, resulting in a negligible volume difference between the dry and wet magmas as shown in Fig. 2a. Then the density difference between the dry and wet magmas at pressures >20 GPa becomes significantly smaller than that at lower pressures, as shown in Fig. 2b. Based on these experimental data, Sakamaki [32] showed that the density of wet magmas containing water <8 wt.% may be greater than that of the preliminary reference Earth model (PREM) at 24 GPa and 1870 K—that is, the density crossover may exist at the bottom of the transition zone along the average temperature profile of the mantle [33].

Dense magmas, however, may not exist at the bottom of the transition zone. Fig. 1 shows the phase and melting relations of the hydrous peridotite (e.g. [13,14]). This figure indicates that the dehydration of slabs occurs at the bottom of the transition zone because of a strong contrast of H_2_O contents between ringwoodite in the transition zone and bridgmanite and ferropericlase in the lower mantle, and dehydration of superhydrous phase B and phase D in the upper part of the lower mantle. Although the wet magmas containing ∼30 wt.% water have a low density relative to the surrounding mantle and move upwards due to buoyancy, continuous descent of the slabs causes dehydration, which can create a low Q and V_s_ region at the boundary between the transition zone and the lower mantle, even in the absence of dense magmas.

The idea that hydrous magmas containing 2∼8 wt.% H_2_O are denser than the surrounding mantle along the normal mantle temperature estimated by Sakamaki [32] may contradict with the melting and phase relations of hydrous CMAS (CaO–MgO–Al_2_O_3_–SiO_2_) pyrolite [13] and those of CFMAS (CaO–FeO–MgO–Al_2_O_3_–SiO_2_) harzburgite [14]. The liquidus curve of pyrolite containing 2 wt.% water locates at 2400–2500 K and the partial melt contains 10 wt.% water at 2300 K at the base of the transition zone as shown in Fig. 1. Thus, the hydrous melts containing 8 wt.% water are formed above 2300 K, suggesting that such hydrous melts cannot exist along the normal mantle geotherm of 1870 K that was proposed by Sakamaki [32]. Zhang *et al.* [14] reported that the partial melt formed by the melting of the hydrous CFMAS harzburgite contains 30 wt.% of water at 20–22 GPa and 1700 K (Fig. 1). Very high temperatures above 2320 K are necessary for the generation of partial melts with 8 wt.% water, assuming hydrous harzburgite [14] has the same liquidus temperature as that of hydrous pyrolite [13]. The phase relations suggest that temperatures ∼400–500 K higher than the normal mantle geotherm are required to produce the hydrous magmas denser than the surrounding mantle at the bottom of the transition zone. Only the hot plumes ascending from the lower mantle would create such high temperatures.

The temperature profile of the lower mantle is uncertain—that is, compositional heterogeneities or stratification in the mantle could provide different temperature profiles to account for the density and velocity distribution of the lower mantle. Separate convection in the upper and lower mantles would create a thermal boundary in the mantle transition zone [34,35]. Counter flows due to the descent of the cold subducting slabs can create the ascent of the hot plumes surrounding the slabs. Therefore, the low Q and Vs region between the transition zone and the lower mantle may be explained by the continuous dehydration and fluid supply due to subduction along the low-temperature slab geotherm. However, we cannot rule out a possibility that the region is caused by the existence of dense hydrous melts at very high temperature due to the ascending plumes from the lower mantle caused by the counter flow generated by slab subduction.

### Stability of hydrous δ-H solid solution, AlO_2_H–MgSiO_4_H_2_

The existence of hydrous minerals in diamond [20,21] supports the locally hydrated mantle transition zone. The descent of the stagnant slabs can carry water farther into the lower mantle [36]. Table 2 summarizes the hydrous phases stable in the lower mantle.

**Table 2.** Hydrous phases stable under the lower-mantle conditions.

Mineral	Formula	Mg/Si	H_2_O wt.%	Reference
Superhydrous phase B = phase C	Mg_10_Si_3_O_12_(OH)_4_	3.3	5.8	[63]
Phase D = phase F = phase G	Mg_1.14_Si_1.73_H_2.81_O_6_	0.66	14.5–18	[63,64]
Phase Egg	AlSiO_3_OH		7.5	[65]
Phase δ	AlO_2_H		15.0	[66]
Phase H	MgSiO_4_H_2_	∼1	15.2	[55]
Phase δ–Η solid solution	(Mg_0.07_Si_0.07_Al_0.86_)O_2_H	∼1	12.8	[50]
Pyrite-type FeO_2_H*_x_*	FeO_2_H*_x_*		*x* = 0.39–1	[39,53]
HH phase	(FeAl)O_2_H*_x_*		*x* ∼ 1?	[58]

The δ-H solid solution AlO_2_H–MgSiO_4_H_2_ is a major carrier of water into the deep lower mantle. Sano *et al.* [25] and Duan *et al.* [37] showed that hydrous δ- AlO_2_H is stable to the bottom of the lower mantle due to a strong O–H bonding caused by the hydrogen-bond symmetrization as discussed below. Recently, Yuan *et al.* [38] reported that the δ-H solid solution containing an FeO_2_H component also is stable up to the base of the lower mantle.

**Figure 3.**
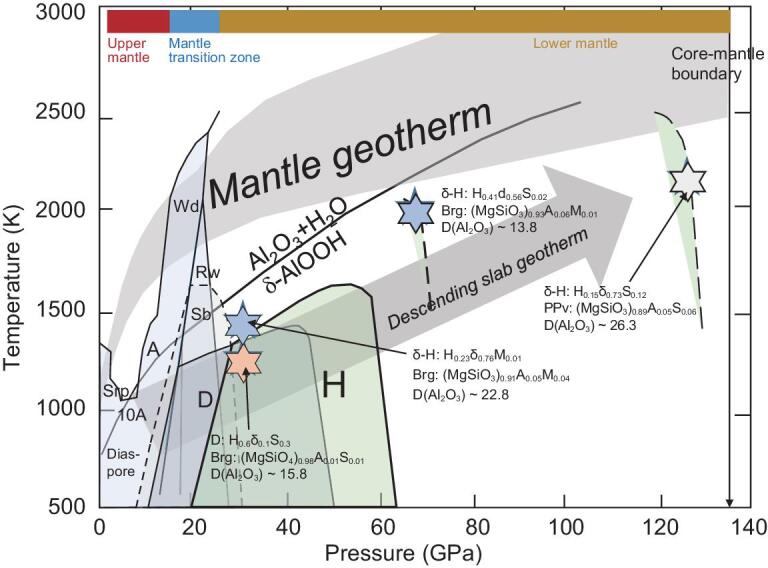
The stability fields of phase H, phase δ, δ-H solid solution, phase D. The compositions of the hydrous phases and coexisting bridgmanite/post-perovskite (ppv) and the partition coefficients of Al_2_O_3_ between the phases, D(Al_2_O_3_), at high pressure and temperature conditions (stars), are given in this figure [59]. The compositions of the coexisting δ-H and bridgmanite/post-perovskite are given as molar fractions of the following components: H, MgSiO_4_H_2_; δ, Al_2_O_4_H_2_; (MgSiO_3_), MgSiO_3_; A, Al_2_O_3_; S, SiO_2_; M, MgO. A gray shaded region and a gray arrow are the normal mantle and the cold slab geotherms, respectively. The stability field of δ-H solid solutions (H_0.23_δ_0.56_Si_0.02_ and H_0.15_δ_0.73_S_0.12_) are shown as broken curves in this figure. Wd, wadsleyite; Rw, ringwoodite; δ-H, hydrous δ-H solid solution; Brg, bridgmanite; PPv, post-perovskite; Sb, superhydrous phase B; D, hydrous phase D; Srp, serpentine; 10A, hydrous 10 Å phase; A, hydrous phase A.

The phase H, MgSiO_4_H_2_ was discovered recently and its stability field has been studied by various authors [39–41]. Fig. 3 shows the stability conditions of hydrous phases stable in the lower mantle such as phase D, phase δ, phase H and δ-Η solid solution. Phase H is stable to 60 GPa and at temperatures <1600 K [40], as shown in this figure. It has an orthorhombic symmetry with a space group Pnnm, the same as that of δ-AlO_2_D and  δ-AlO_2_H at >10 GPa [42].

The coexistence of bridgmanite/post-perovskite and δ-H solid solution can modify the alumina contents of bridgmanite and post-perovskite, the major minerals in the lower mantle. Fluids dehydrated at the top of the lower mantle react with aluminous bridgmanite along the geotherms of the slab and normal mantle to create alumina-depleted bridgmanite coexisting with the phase δ-H solid solution. The partition coefficient of alumina D_δ/Brg or PPv_ is very large, at ∼14–26, in a wide pressure and temperature range. The Al_2_O_3_ contents of bridgmanite in MORB and peridotite under the dry condition are ∼15 and ∼5 wt.%, respectively [43], whereas the Al_2_O_3_ contents decrease significantly to ∼3 and ∼1 wt.%, respectively, by the addition of water and the formation of the hydrous phase due to a large partition coefficient of alumina between hydrous δ-H solid solution and bridgmanite.

Recent works on the stability of δ-phase in the MFASH (MgO–Fe_2_O_3_–Al_2_O_3_–SiO_2_–H_2_O) system showed the stability of the δ-phase in an iron-bearing system in the lower mantle and a strong partitioning of Al_2_O_3_ into the iron-bearing δ-phase coexisting both with bridgmanite and the post-perovskite phase [38]. Depletion of Al_2_O_3_ in bridgmanite and post-perovskite under the wet conditions provides effects on mantle dynamics due to the modification of the phase relations—that is, the Al_2_O_3_ depletion in bridgmanite lowers the pressure of the garnet–perovskite transition in MORB [43]. The depletion of Al_2_O_3_ also lowers the post-perovskite transition pressure and sharpens the phase transition at the base of the lower mantle.

### Hydrogen-bond symmetrization in high-pressure hydrous phases

Hydrous phases such as phase δ [44] and phase D [45], stable in the lower mantle, have a strong and stable O–H bonding under high-pressure conditions because of hydrogen-bond symmetrization.

**Figure 4.**
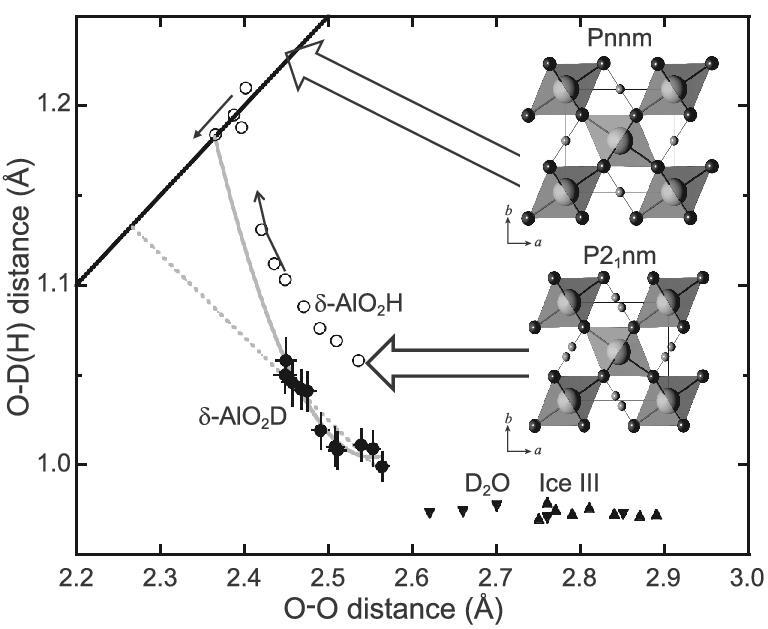
The evolution of the O-D(H) distance due to compression as a function of the O–O distance of δ-AlO_2_D (solid circles) [68], δ-AlO_2_H (open circles) [69] and Ice III (solid triangles) [70,71]. The change for the two space groups with δ-AlO_2_D(H), P2_1_nm and Pnnm, are also shown in the figure. Open circles indicate the change in O–O and O–H due to compression for the phase transition from P2_1_nm to Pnnm occurs at high pressure. The thin arrows indicate the direction of the change due to compression. The solid bold line indicates the symmetrization line where the O–O distance is equal to twice the O–D distance. The dotted gray line and solid gray curve indicate the extrapolations using linear and second-order functions, respectively (modified from Sano-Furukawa *et al.* [68]).

Because of this bonding nature, the hydrous phases become stable and show elastic hardening at high pressure. The relation between O–H and O–O bond lengths due to the symmetrization of the O–H bond is shown in Fig. 4. The hydrogen-bond symmetrization has been observed in polymorphs of water ice. An inflection in the compression curves expressed by the Vinet equation of the state has been reported by Wolanin *et al.* [46] and Pruzan *et al.* [47] at 66 GPa for H_2_O and 84 GPa for D_2_O. This inflection can be explained by proton disordering—a signature of hydrogen-bond symmetrization.

Elastic hardening due to hydrogen-bond symmetrization has been observed by Tsuchiya *et al.* [45] and Hushur *et al*. [48] in hydrous phase D. They reported an increase in the bulk modulus, K_T_, from 173(2) to 212(15) GPa for hydrous phase D at ambient temperature due to hydrogen-bond symmetrization.

Tsuchiya *et al.* [49] showed by *ab initio* calculations that both the P- and S- velocities of δ-AlO_2_H increase with increasing pressures to the mantle transition-zone conditions, becoming faster than those of wadsleyite, ringwoodite and majorite, in the mantle transition zone, and are comparable to those of bridgmanite at the shallow lower mantle. A strong O–H bond due to the hydrogen-bond symmetrization accounts for a high sound velocity of δ-AlO_2_H.

A high sound velocity of δ-AlO_2_H comparable to bridgmanite by the hydrogen-bond symmetrization has strong implications for the role of water in the lower mantle. Phase H, MgSiO_4_H_2_ and phase H-δ solid solution are considered to have high seismic velocities comparable to bridgmanite because of a structural similarity to δ-AlO_2_H. Thus, it might be difficult to detect the existence of these hydrous phases in the shallow lower mantle by seismology.

### Iron–water reaction and the role of hydrous phases at the core–mantle boundary (CMB)

**Figure 5.**
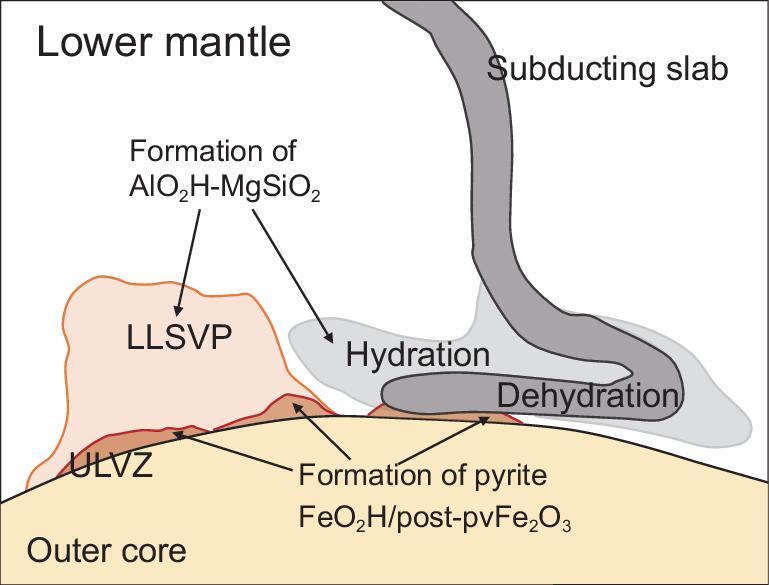
The cartoon of the core–mantle boundary, indicating dehydration of slabs and hydration of surrounding mantle. δ-H solid solution AlO_2_H–MgSiO_4_H_2_ may exist at the LLSVP (large low shear velocity province). The ULVZ (ultralow velocity zone) may be caused by existence of hydrous phase FeO_2_H and PPv-Fe_2_O_3_.

In the previous sections, we learned that hydrous phase δ-H solid solution, AlO_2_H–MgSiO_4_H_2_, is the most important carrier of water in the slabs descending into the lower mantle [25,50]. This phase is expected to be dehydrated to produce fluids due to a steep geothermal profile at the CMB. Fluids generated by this process can hydrate the surrounding mantle at the base of the lower mantle and hydrated regions may create LLSVP (large low shear velocity province). The dehydration of δ-AlO_2_H occurs at ∼2400 K [25,37] at the base of the lower mantle, and dissolution of phase H component (MgSi)O_4_H_2_ further lowers the decomposition temperature [40], whereas pyrite FeO_2_H seems to decompose to post-perovskite Fe_2_O_3_ and fluid at higher temperatures >2500 K [39], although the phase boundary is not yet determined precisely. Thus, the fluids generated by the decomposition of the δ-H solid solution may react with iron from the core to create an iron hydrate FeO_2_H*_x_*, which can cause the seismic anomaly of the ULVZ (ultralow velocity zone), as was suggested by Liu *et al.* [51] and Yuan *et al.* [52]. The processes expected at the bottom of the mantle are schematically shown in Fig. 5.

Pyrite structure FeO_2_ (*Pa*${\rm{\bar{3}}}$) was recently discovered as a reaction product between hematite and oxygen under the condition of 76 GPa and 1800 K [53]. Hu *et al.* [54] reported the formation of pyrite-type FeO_2_H*_x_* based on their experimental results with scattered unit-cell volume values of pyrite FeO_2_H*_x_* at a constant pressure and *ab initio* calculation. Nishi *et al.* [39] also reported FeO_2_H pyrite by experiments and theoretical calculations suggesting the phase is close to stoichiometric, *x* ∼ 1.

Yuan *et al*. [52] reported that the iron–water reaction produces FeO and FeH*_x_* <78 GPa, whereas FeO_2_H*_x_* and FeH*_x_* (hcp and dhcp) were formed above 78 GPa at ∼2000 K. The volumes of FeO_2_H*_x_* reported by Yuan *et al.* [52] was comparable to that of stoichiometric pyrite FeO_2_H [55]—that is, the formation of nearly stoichiometric pyrite FeO_2_H*_x_* with *x* ∼ 1. Liu *et al.* [51] identified the iron–water reaction to form pyrite FeO_2_H*_x_* and fcc-FeH*_x_* after quenching at 86 GPa.

The seismic velocities, V_P_, V_s_ and V_φ_, are significantly smaller than those of the lower-mantle minerals such as ferropericlase, bridgmanite and post-perovskite [51,52]. V_φ_ of pyrite FeO_2_H*_x_* is 10% smaller than that of PREM [56] at the bottom of the mantle. Therefore, it may be a candidate for the ULVZ [52,53]. The high-pressure polymorph of Fe_2_O_3_ with a post-perovskite structure can be formed by the dehydration of pyrite FeO_2_H*_x_* [39]. Therefore, post-perovskite Fe_2_O_3_ also can be a candidate phase for the ULVZ. Decomposition of pyrite FeO_2_H*_x_* at the bottom of the mantle because of its dynamic instability could create global geological events, such as the Great Oxidation Event [57]. Recently, a new high-pressure form of AlO_2_H–FeO_2_H solid solution with a hexagonal symmetry has been reported at the base of the lower mantle [58]. This phase might be a potential water reservoir at the CMB regions. Further studies should be conducted to confirm the reservoir phases of water and to test the hypothesis of the generation of H_2_ and O_2_ at the bottom of the mantle [57].

## CONCLUSIONS

Seismic and electrical conductivity observations combined with experimental mineral physics data on the sound velocity and electrical conductivity of minerals suggest the transition zone that is hydrated at least locally (e.g. [22]). Continental and oceanic sediment components together with the basaltic and peridotite components might be stored in the mantle transition zone (e.g. [19]). Low seismic velocity regions have been reported at ∼410 km beneath some plate convergent regions (e.g. [9]). These regions might be caused by the existence of dense, volatile-rich magmas.

Water can be carried farther into the lower mantle by the descent of the slabs due to gravitational instability. The anomalous Q and V_s_ regions might be created at the top of the lower mantle. Dehydration from the slabs produces fluids or hydrous melts in this region due to a large difference in the water solubility between the transition zone and the lower mantle assemblages (e.g. [59]). Although hydrous magmas without density crossover can escape upwards, continuous descent of the slabs causes dehydration from the slabs and produces low Q and V_s_ regions at the shallow part of the lower mantle. δ-H solid solution AlO_2_H–MgSiO_4_H_2_ is a major carrier of water into the lower mantle. The hydrogen-bond symmetrization could occur in various hydrous phases stable in the mantle (e.g. [44]).

The CMB is a region where extensive reaction between water and iron might occur. The δ-H solid solution is stable to the CMB conditions. Therefore, this hydrous phase carries water into the base of the lower mantle and also into the core. Pyrite FeO_2_H*_x_* can be formed due to a reaction between the core and hydrated slabs at the CMB. This phase could be a potential candidate existing at the ULVZ [51,52]. Formation of FeO_2_H*_x_* and its decomposition due to its thermal instability at the CMB could cause global geodynamical events [57].
